# Association of Bioavailable 25(OH)D Levels With Endometriosis in Infertile Bangladeshi Women: A Cross‐Sectional Study

**DOI:** 10.1002/hsr2.71070

**Published:** 2025-07-18

**Authors:** Ratna Paul, Mst Sabrina Moonajilin, Hridhima karmakar, Himel Pal, Swatilekha Paul, Sujit Kumar Sarker, Labina Taher

**Affiliations:** ^1^ Dhaka Medical College and Hospital Dhaka Bangladesh; ^2^ Department of Public Health and Informatics Jahangirnagar University Savar Bangladesh; ^3^ Viqarunnesa Noon School and College Dhaka Bangladesh; ^4^ Holy Cross Girls' High School Tejgaon Bangladesh

**Keywords:** Bangladesh, bioavailable 25(OH)D, endometriosis, infertility, women

## Abstract

**Background and Aims:**

Infertility is a significant public health issue worldwide, and endometriosis is a major contributor to infertility, impacting 6%–10% of reproductive‐aged women. The objective of the study is to assess the relationship between bioavailable 25(OH)D (BVD) levels and endometriosis among infertile women.

**Methods:**

A cross‐sectional study was carried out among infertile women at Dhaka Medical College Hospital in Bangladesh, examining the association of bioavailable 25(OH)D levels with endometriosis in infertile Bangladeshi women.

**Results:**

A statistically significant difference in BVD levels was observed between infertile women with and without endometriosis. Specifically, the mean BVD level was substantially higher in infertile women without endometriosis compared to those diagnosed with the condition. BVD levels were significantly lower among the participants aged between 26 and 30 years for women without endometriosis. Women aged 26–30 years had the highest BVD, while those aged 31–35 years had the lowest. The association between body mass index (BMI) and BVD levels is statistically significant in women with endometriosis. Notably, underweight women had the highest mean BVD, followed by obese women, while overweight and normal BMI participants had notably lower values. Rural women with endometriosis showed slightly higher BVD levels than urban women, though this was not statistically significant. In the non‐endometriosis group, urban women had lower BVD levels than their rural counterparts. BVD levels show slight variations across educational levels, but without significant trends. However, we did not find any significant association between BVD levels and other variables.

**Conclusion:**

This study adds to the growing body of evidence linking BVD with infertility, particularly in the context of endometriosis. While consistent with global trends, the unique socio‐demographic and cultural factors in Bangladesh highlight the need for region‐specific approaches to address vitamin D deficiency and its reproductive health implications.

## 1 Introduction

Infertility is a significant public health issue worldwide and affects approximately 10%–15% of couples, with female factors contributing significantly to this burden [1]. Endometriosis, a chronic disorder impacting 6‐10% of reproductive‐aged women, is a major contributor to infertility [2]. This condition is marked by the presence of endometrial tissue outside the uterus, which hinders ovarian function, implantation, and the maintenance of early pregnancy [3]. Endometriosis is a prevalent condition among infertile women, impacting fertility through various mechanisms and at multiple levels [4]. Previous research indicates it affects approximately 25%–50% of women experiencing infertility, and is present in 30% to 50% of patients with endometriosis who encounter challenges with conception [5]. Fecundity in healthy couples ranges from 0.15 to 0.20 each month and declines with age; endometriosis‐affected women often have lower monthly fecundities of 0.02–0.1 per month [6, 7]. Endometriosis is also linked to a reduced live birth rate, driven by mechanisms such as distorted pelvic anatomy, endocrine or ovulatory dysfunction, impaired peritoneal function, and disrupted hormonal or cell‐mediated processes within the endometrium [7].

Inflammatory reactions, immunological reactions, cell growth and death, angiogenesis, and other processes can influence endometriosis. It has been suggested that the disease's complex pathophysiology may involve an immune system malfunction that results in a persistent inflammatory state [8]. Inhibiting inflammation, controlling angiogenesis, and contributing to the pathophysiology of endometriosis are among the tasks of the vitamin D system [9]. The bioavailability of 25‐hydroxyvitamin D [25(OH)D] is influenced by serum albumin levels and variations in vitamin D binding protein (DBP). Serum 25‐hydroxyvitamin D [25(OH)D] is widely recognized as the primary biomarker for evaluating vitamin D status in clinical and research settings. However, total serum 25(OH)D does not always reflect the biologically active fraction of the vitamin. The term “bioavailable vitamin D” (BVD) refers to the portion of circulating 25(OH)D that is not tightly bound to vitamin d‐binding protein (DBP) [10]. BVD is considered a more accurate reflection of tissue availability and function, particularly in pathological states such as endometriosis [11].

Studies show that women's reproductive health is influenced by their BVD status, considering that BVD has immunomodulatory, anti‐inflammatory, and antiproliferative properties that can contribute to the pathogenesis of endometriosis [8, 12]. Vitamin D, a fat‐soluble vitamin crucial for human health, is primarily obtained through dietary sources, supplements, and skin synthesis via exposure to ultraviolet B radiation. It plays a vital role in calcium and phosphorus metabolism, as well as maintaining bone health [13]. Research has expanded its significance to include immunomodulation, regulation of inflammatory responses, and cellular differentiation, processes that are integral to reproductive health [14, 15]. Vitamin D receptors (VDR) are found in key reproductive tissues, including the ovary, endometrium, and placenta, highlighting their essential role in fertility [16].

Endometriosis' pathogenesis may be influenced by vitamin D's immunologic actions and the development of the VDR in reproductive organs [17]. The potential involvement of BVD is becoming increasingly interesting. Over the past 20 years, several studies have investigated the association between endometriosis and BVD levels, but the findings have been mixed [18, 19]. Since the human endometrium encompasses the 1‐hydroxylase (CYP27B1) and VDR, it may serve as a location for the external synthesis and potential action of vitamin D [20]. The BVD functions through the VDR and has been shown to induce the destruction of microbial agents while suppressing antigen presentation and dendritic cell maturation [21]. In addition, vitamin D inhibits the proliferation of lymphocytes, particularly Th1 cells, which encourages the conversion of Th1 cells to Th2 cells [22]. With the presence of BVD and its receptors in the ovaries, the production of steroid hormones progesterone and estrogen increases [23].

Vitamin D deficiency is highly prevalent in South Asia, including Bangladesh, despite abundant sunlight, due to factors such as limited sun exposure, cultural practices, and dietary insufficiencies [24]. Studies have linked low BVD levels to adverse reproductive outcomes, including ovulatory dysfunction and reduced endometrial receptivity [25, 26]. In endometriosis, BVD's immunomodulatory and anti‐inflammatory properties may be crucial in mitigating disease progression and improving fertility outcomes [27, 28, 29]. In South Asia, including Bangladesh, the prevalence of noncommunicable diseases (NCDs) and micronutrient deficiency has risen in recent decades and is responsible for over 60% of all deaths in the region [30, 31, 32, 33]. NCDs place a tremendous financial strain on the already overburdened healthcare systems in many LMICs, including Bangladesh. The rising burden of NCDs is especially concerning for reproductive‐age women. In Bangladesh, socio‐cultural norms, environmental conditions, and limited access to healthcare complicate infertility management as well as motherhood [34, 35].

Low BVD is highly prevalent among fertile women in Bangladesh, with reported rates of approximately 64% in rural populations and 63% in urban areas such as Dhaka [36, 37, 38]. There is a paucity of research on the relationship between BVD status and infertility, particularly in women with and without endometriosis. This study seeks to address this gap by evaluating BVD among Bangladeshi infertile women and exploring socio‐demographic and clinical factors that may influence. By shedding light on this association, the findings aim to inform targeted interventions to improve fertility outcomes in resource‐constrained settings. We hypothesized that endometriosis and BVD levels among infertile women of reproductive age are related. This study aimed to assess BVD levels in infertile women with and without endometriosis and identify the relationship between BVD levels and endometriosis by comparing these two groups of infertile women.

## 2 Materials and Methods

### 2.1 Study Design and Data Collection Procedure

A cross‐sectional study was carried out at the Reproductive Endocrinology and Infertility Unit in Dhaka Medical College Hospital (DMCH) in Bangladesh during the COVID‐19 pandemic. A structured and pretested, interviewer‐administered questionnaire was used to collect data between January 2020 and October 2020 concerning sociodemographic, infertility, and BVD‐related data. Participants were selected by using a convenience sampling technique. Data and samples were collected from those who voluntarily agreed to join the study and had given written consent.

### 2.2 Inclusion and Exclusion Criteria

The inclusion criteria for the participants included infertile women of reproductive age living in Bangladesh who visited the Reproductive Endocrinology and Infertility Unit at DMCH. Participants with celiac disease or other causes of malabsorption, kidney diseases, receiving hormonal therapy (GnRHa, progesterone, or combined estrogen‐progesterone), medications affecting bone metabolism, receiving Vitamin D or calcium supplementation, and the presence of chronic disease were excluded from the study.

### 2.3 Sample Size and Sampling Technique

The sample size was calculated based on the cumulative prevalence of 39%, 5% precision, and 95% confidence interval using the following formula:

$$n=\frac{Z^{2}p(1-p)}{e^{2}},$$

here *n* = required sample size; *p* = prevalence of infertile couples (15%) = 0.15; *q* = (1 − *p*) = 1 − 0.15 = 0.85; *Z* = *Z* value at 5% level of significance = 1.96; e = margin of error = 5% = 0.05. Using this formula, the estimated sample size was 196. However, we included 101 participants by applying convenience sampling techniques due to the COVID‐19 pandemic and the accessible population for this specific group of women with infertility and endometriosis was relatively limited.

### 2.4 Ethics Approval and Consent to Participate

The study was carried out following the guidelines of the Declaration of Helsinki of 2013. The ethical aspect of the current study was approved by the ethical review committee of DMCH (ERC‐DMC/ECC/2019/380(R)). Participants were free to withdraw from the study at any time and at any stage. Written informed consent was obtained from each participant after explaining the aim and purpose of the research. Confidentiality was guaranteed, and anonymity was preserved; no participant was identified in any report or publication resulting from this study [39].

### 2.5 Measures

#### 2.5.1 Socio‐Demographic Variables

Socio‐demographic data of the participants were collected through closed‐ended questions involving their age, level of education, occupation, residence area, religion, and monthly family income (upper class: monthly income > 40,000 TK, middle class: monthly income 10,000–40,000 TK, and lower class: monthly income 10,000 TK).

#### 2.5.2 Anthropometric Measurement

The body weight and height of each participant were measured using standard calibrated scales and a nonstretchable tape affixed to a flat vertical surface, respectively, with precision to the nearest 0.1 kg and 0.5 cm. BMI was calculated with the formula weight (kg)/height (m^2^). Then it was categorized into four groups following World Health Organization (WHO) criteria (BMI < 18.5 kg/m^2^ = underweight, 19–24.99 kg/m^2^ = normal, 25–29.9 kg/m^2^ = overweight, and ≥ 30 kg/m^2^ = obese) [33].

#### 2.5.3 Infertility‐Related Variables

Infertility‐related data were collected concerning the type of infertility (either primary or secondary), the presence of acne or hirsutism, and their body weight status.

#### 2.5.4 Endometriosis Evaluation

Endometriosis was confirmed by laparotomy or laparoscopy at the Reproductive Endocrinology and Infertility Unit at DMCH, consistent with global diagnostic standards [40].

#### 2.5.5 BVD Evaluation in the Laboratory

To assess BVD status, 5 mL of venous blood was collected from each participant under aseptic conditions and the direct supervision of the principal investigator. Samples were centrifuged within 30 min of collection to separate serum, which was then stored at −20°C until analysis. A serum total of 25(OH)D concentrations were measured using a chemiluminescence immunoassay (CLIA), and DBP levels were measured via ELISA. Due to the limited availability of BVP assays in clinical laboratories in Bangladesh, researchers used estimation formulas based on total 25(OH)D, albumin, and DBP. The equation we used is based on the established formula described by Bikle et al. [41]. Details of the BVD estimation, including individual values for total 25(OH)D, DBP, albumin, and calculated BVD, are provided in Supplementary File (S1). The blood test was tested at the Institute of Nuclear Medicine and Allied Science at DMCH and the Biochemistry Department. The level of BVD was categorized into the following groups: (Severe deficiency: less than 12 ng/mL; Moderate deficiency: 12–20 ng/mL; Insufficiency: between 21 and 29 ng/mL; Sufficient: higher than 30 ng/ml) [42]. These categories are based on the concentration of 25(OH)D in the blood, which is the primary circulating form of vitamin D.

### 2.6 Data Analysis

All data were coded and analyzed using two statistical software packages (Microsoft Excel 2019 and IBM SPSS Statistics version 25.0). Microsoft Excel was used for data cleaning, coding, editing, and sorting. Then the data set was imported into the SPSS software. Descriptive statistics (e.g., frequency, percentage, means, and standard deviations) and inferential statistics (e.g., *t*‐test, one‐way ANOVA) were used to analyze the data. However, a *t*‐test was done to figure out the association between BVD levels and endometriosis. In addition, *t*‐test or one‐way ANOVA were also performed to determine the influencing factors for vitamin D deficiency, where the *p* value of < 0.05 was used for all statistical significance.

## 3 Results

### 3.1 Socio‐Demographic Characteristics of Study Participants

Among the total participants (*n* = 101), 50.7% (*n* = 51) of women had infertility without endometriosis, while 49.3% (*n* = 50) had infertility with endometriosis. Table 1 shows the socio‐demographic characteristics of the study participants distributed by two groups (infertile women without endometriosis and those with endometriosis). Among both groups, most of the participants were from the age group 18–25 years, where the mean age of the participants was 26.53 (SD = 5.68) years. In addition, 8.0% of participants with endometriosis were more than 35 years old, compared to only 5.9% of participants without endometriosis. Urban residence is more prevalent among patients without endometriosis (86.3%) compared to those with endometriosis (66%). A greater proportion of women with endometriosis belong to the low socioeconomic group (56%) compared to those without (41.2%). Educational attainment is generally lower in women with endometriosis, with 64% below the SSC level compared to 54.9% in the group without endometriosis. Hirsutism and acne are slightly more common in women with endometriosis (14% for both conditions) compared to those without (9.8% and 11.8%, respectively) (Table 1).

**Table 1:** Socio‐demographic characteristics of study participants (Infertility patients without endometriosis and with endometriosis).

Variables	Infertility without endometriosis (*N* = 51)		Infertility with endometriosis (*N* = 50)	
	Frequency	Percentages	Frequency	Percentages
Age				
18–25	21	41.2	27	54.0
26–30	13	25.5	6	12.0
31–35	14	27.5	13	26.0
> 35	3	5.9	4	8.0
Occupation				
Housewife	43	84.3	42	84.0
Service	8	15.7	8	16.0
Residence				
Urban	44	86.3	33	66.0
Rural	7	13.7	17	34.0
Religion				
Muslim	50	98.0	46	92.0
Hindu	1	2.0	4	8.0
Socioeconomic status				
Low	21	41.2	28	56.0
Middle	26	51.0	20	40.0
High	4	7.8	2	4.0
Educational status				
Below SSC	28	54.9	32	64.0
SSC	6	11.8	7	14.0
HSC	5	9.8	5	10.0
Graduate or above	12	23.5	6	12.0
Presence of hirsutism				
Yes	5	9.8	7	14
No	46	90.2	43	86
Presence of acne				
Yes	6	11.8	7	14
No	45	88.2	43	86

### 3.2 BVD Level in Infertile Women With and Without Endometriosis

**Figure 1:**
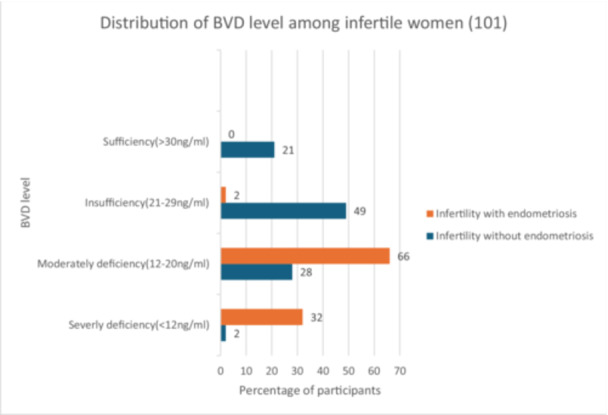
Different categories of BVD levels in infertility patients without endometriosis and infertility patients with endometriosis.

Different categories of BVD levels in infertile women without endometriosis and infertile women with endometriosis are presented in Figure 1. Severe and moderate levels of BVD deficiency were tremendously higher among infertile women with endometriosis, and vice versa regarding insufficiency and sufficiency. Among infertile women without endometriosis, about 2.0% had severe BVD deficiency, 28% had a moderate deficiency, 49.0% had insufficiency, and 21% had a sufficient level of BVD, whereas, among infertile women with endometriosis, 32.0% had severe BVD deficiency, 66% had moderate deficiency, 2.0% had insufficiency, and none had a sufficient level of BVD.

### 3.3 Mean BVD Score Among Women With and Without Endometriosis

The mean comparison of BVD levels in infertile women without endometriosis and infertile women with endometriosis is shown in Table 2. A significant difference in BVD levels has been observed between infertile women without endometriosis and infertile women with endometriosis (*t* = 164.949, *p* < 0.001). In addition, the mean BVD level was considerably higher among infertile women without endometriosis (26.95 ± 6.78 vs. 11.71 ± 2.69). Table 3 shows the difference in BVD levels between infertile women without endometriosis and those with endometriosis, along with the risk factors. BVD levels were significantly lower among the participants aged between 26 and 30 years for women without endometriosis (*p* = 0.031). Women aged 26–30 years had the highest BVD (mean = 27.50 ng/mL), while those aged 31–35 years had the lowest (mean = 20.56 ng/mL). The association between BMI and BVD levels is statistically significant in women with endometriosis (*p* = 0.02). Notably, underweight women had the highest mean BVD (14.41 ng/mL), followed by obese women (12.40 ng/mL), while overweight and normal BMI participants had notably lower values. Rural women with endometriosis showed slightly higher BVD levels than urban women (12.25 vs. 11.43 ng/mL), though this was not statistically significant (*p* = 0.31). In the non‐endometriosis group, urban women had lower BVD levels than rural counterparts. BVD levels show slight variations across educational levels, but without significant trends. However, we did not find any significant association between BVD levels and other variables.

**Table 2:** Comparison of mean BVD levels in infertility patients without endometriosis and infertility patients with endometriosis.

Type	Minimum	Maximum	Mean	SD	*t*	*p* value
Infertility without endometriosis	7.89	38.42	26.95	6.78	164.94	< 0.001
Infertility with endometriosis	7.44	22.70	11.71	2.69		

Abbreviation: SD, standard deviation.

**Table 3:** Comparison of BVD levels among the participants (with and without endometriosis).

Variables	BVD level							
	Infertility without endometriosis				Infertility with endometriosis			
	Mean	SD	F/t	*p* value	Mean	SD	F/t	*p* value
Age								
18–25	26.31	7.06	3.222	0.031	11.31	3.26	0.632	0.59
26–30	27.50	4.43			12.11	1.54		
31–35	20.56	6.32			12.49	1.34		
> 35	24.85	8.73			11.26	3.19		
Occupation								
Housewife	23.29	7.21	0.565	0.456	11.34	4.80	0.179	0.67
Service	25.26	6.74			11.78	2.16		
Residence								
Urban	24.61	6.80	0.791	0.378	11.43	2.95	1.030	0.31
Rural	27.07	6.77			12.25	2.05		
Religion								
Muslim	24.98	6.84	0.046	0.831	11.54	2.74	2.358	0.13
Hindu	23.50	1.01			13.67	1.87		
Socioeconomic status								
Low	24.12	7.77	0.276	0.760	11.85	3.12	.227	0.79
Middle	25.61	6.49			11.43	2.15		
High	24.98	1.74			12.53	.374		
Educational status								
Below SSC	25.21	7.50	0.261	0.853	12.36	2.91	2.256	0.09
SSC	26.40	5.26			10.18	1.53		
HSC	25.09	5.27			11.62	2.08		
Graduate or above	23.56	6.67			10.09	1.70		
Presence of hirsutism								
Yes	24.55	2.25	0.002	0.969	26.62	5.03	2.350	0.13
No	24.69	7.04			24.45	6.94		
Presence of acne								
Yes	26.62	5.03	0.456	0.503	11.82	1.22	.019	0.89
No	24.45	6.94			11.65	2.90		
Types of infertility								
Primary	26.41	6.79	3.175	0.081	11.85	3.04	.288	0.59
Secondary	22.99	6.57			11.41	1.99		
BMI category								
Underweight	—	—	2.599	0.085	14.41	.957	3.571	0.02
Normal	23.77	7.50			10.83	4.29		
Overweight	24.45	5.72			10.80	1.51		
Obese	29.76	4.87			12.40	1.98		

## 4 Discussion

This study found that infertile women with endometriosis had significantly lower BVD levels. The findings of this study align with the results of other studies [28, 43], who also reported significantly reduced BVD levels among women with endometriosis compared to healthy controls. These findings are consistent with a previous study that reported that women with endometriosis had lower BVD serum levels than disease‐free controls [18]. These findings support emerging evidence that vitamin D, particularly in its bioavailable form, may play a role in modulating inflammatory pathways, immune responses, and endometrial receptivity, all of which are disrupted in endometriosis [44]. The mean BVD levels in this study were lower than those reported in similar populations globally, reflecting the high prevalence of BVD deficiency in South Asia. A study found this regional trend due to factors such as limited sun exposure due to cultural clothing practices, inadequate dietary intake of vitamin d‐rich foods, and insufficient public health initiatives [45].

Rural women with endometriosis showed slightly higher BVD levels than urban women. While not statistically significant, rural women may benefit from greater sun exposure, which enhances vitamin D synthesis [46]. However, the overall weak associations suggest that pathological processes in endometriosis may override socio‐behavioral factors in determining BVD levels. These cultural and environmental factors likely contribute to the widespread deficiency observed in Bangladeshi women. Urban residents without endometriosis exhibited higher BVD levels compared to rural residents, consistent with findings by Griffin et al. [47], who noted that rural populations often have less access to fortified foods and supplements. Similarly, patients in lower Socioeconomic groups showed greater vitamin D deficiency, reflecting economic barriers to obtaining adequate nutrition and healthcare access [48].

Among women without endometriosis, age appears to influence BVD levels more prominently. In contrast, among women with endometriosis, the uniformly low BVD levels may reflect a pathological suppression of BVD regardless of age. These patterns suggest that age‐related metabolic or hormonal changes could moderate BVD status only in the absence of inflammatory pathology. BVD levels declined with increasing age and higher BMI, with statistical significance noted in the endometriosis group. Miao et al. [49] observed a similar relationship, attributing reduced BVD in obesity to sequestration of the vitamin in adipose tissue. Age‐related declines are likely due to decreased skin synthesis capacity and dietary intake over time. The association between BMI and BVD levels is statistically significant in women with endometriosis in this study. The relationship between BMI and BVD in endometriosis patients is complex. Adipose tissue can sequester BVD, but chronic inflammation (more common in higher BMI and endometriosis) may alter DBP and albumin levels, affecting BVD calculation. The unusually high BVD in underweight individuals might be linked to altered protein‐binding dynamics in low‐fat environments [50]. These findings underscore the importance of tailored interventions addressing age and weight management to improve vitamin D status.

This study found no significant differences in BVD levels based on education or type of infertility (primary vs. secondary). Bayati et al. [15] reported a positive correlation between higher educational attainment and better health literacy, resulting in improved nutritional awareness and practices. The lack of significant association in this study may reflect specific cultural and healthcare access patterns in Bangladesh. Intervention studies have shown that correcting vitamin D deficiency through supplementation may improve reproductive outcomes [25]. Lower BVD levels in women with endometriosis support the hypothesis that insufficient BVD may contribute to immune dysregulation, chronic inflammation, and tissue remodeling associated with endometriosis. As BVD has known immunomodulatory and antiproliferative properties, its deficiency may exacerbate endometrial lesion formation and related infertility [51]. Socioeconomic and educational disparities may influence BVD status and infertility outcomes, with patients in lower socioeconomic groups generally exhibiting lower levels. While this study did not include supplementation data, the low mean levels observed suggest a significant gap in addressing vitamin D deficiency. Given the high prevalence of vitamin D deficiency in Bangladesh and the potential link with reproductive health, our findings support the need for targeted public health interventions. Vitamin D supplementation, nutritional education, and policies to enhance safe sun exposure should be prioritized for socioeconomically disadvantaged women with infertility.

## 5 Limitations

This study has several limitations. First, as this was a cross‐sectional study, we cannot determine whether low vitamin D levels are a cause or consequence of endometriosis. Longitudinal cohort studies and randomized controlled trials are needed to investigate the temporal sequence and causal pathways. Second, measuring BVD levels only once, without follow‐up assessments, may not accurately represent the BVD status of women over time, particularly in the context of infertility. Third, the study did not consider the stage of endometriosis and was conducted in a single medical facility, which might limit the external validity of the findings to other healthcare settings or regions. Fourth, this study excluded individuals with certain medical conditions, hormonal therapies, and chronic diseases. This might result in a sample that does not fully represent the diversity of infertile women or those with certain conditions that could interact with the variables under investigation. Fifth, this study did not assess several important confounders that may influence BVD, such as dietary intake, sunlight exposure, skin pigmentation, physical activity, and seasonal variation. Sixth, this study was based on a relatively small sample from a single tertiary hospital in Dhaka, which may limit the generalizability of the findings. Future studies should incorporate larger, multicentric populations to enhance external validity. This study utilized convenience sampling to select participants, which may introduce selection bias. The sample might not be representative of the broader population of infertile women in Bangladesh, limiting the generalizability of the findings to a wider context. Finally, all cases of endometriosis were confirmed via laparoscopy and clinical signs/symptoms, staging data were not available in a consistent format and were therefore not included in analysis. Comparative studies assessing vitamin D status across different stages of endometriosis are warranted.

These factors should be considered in future studies, along with other factors related to BVD. To determine the efficacy of vitamin D supplementation in improving fertility outcomes among women with endometriosis, longitudinal studies and randomized controlled trials are necessary. Furthermore, investigating the genetic and environmental factors influencing BVD in this population could provide additional insights.

## 6 Conclusion

Current findings demonstrated that BVD circulating levels were lower among infertile women with endometriosis, seeking medical help for a couple's infertility. Since BVD levels are significantly associated with endometriosis, it can be considered a risk factor. Addressing vitamin D deficiency, particularly in socio‐economically disadvantaged and endometriosis‐affected populations, may play a crucial role in improving infertility outcomes. This study adds to the growing body of evidence linking BVD with infertility, particularly in the context of endometriosis. While consistent with global trends, the unique socio‐demographic and cultural factors in Bangladesh highlight the need for region‐specific approaches to address BVD and its reproductive health implications. Future research should focus on longitudinal studies and interventional trials to establish causal relationships and evaluate the efficacy of vitamin D supplementation in improving fertility outcomes.

## Recommendations

- Implement vitamin D supplementation programs for infertile women, particularly those with endometriosis.
- Increase awareness about the importance of vitamin D for reproductive health among at‐risk groups.
- Develop tailored support programs for socio‐economically disadvantaged populations to improve overall health outcomes.

## Author Contributions

**Ratna Paul:** conceptualization, methodology, writing – review and editing, data curation. **Mst Sabrina Moonajilin:** conceptualization, methodology, data curation, writing – original draft, writing – review and editing. **Hridhima karmakar:** data curation, writing – review and editing. **Himel Pal:** data curation, writing – review and editing. **Swatilekha Paul:** data curation, writing – review and editing. **Sujit Kumar Sarker:** data curation, writing – review and editing. **Labina Taher:** data curation, writing – review and editing.

## Conflicts of Interest

The authors declare no conflicts of interest.

## Transparency Statement

The lead author Mst Sabrina Moonajilin affirms that this manuscript is an honest, accurate, and transparent account of the study being reported; that no important aspects of the study have been omitted; and that any discrepancies from the study as planned (and, if relevant, registered) have been explained.

## Supporting information

S1 Vitamine D.

## Data Availability

The data that support the findings of this study are available on request from the corresponding author. The data are not publicly available due to privacy or ethical restrictions.
