# Supplementary material for: Association of Bioavailable 25(OH)D Levels With Endometriosis in Infertile Bangladeshi Women: A Cross‐Sectional Study
Source: Health Sci Rep. 2025 Jul 18;8(7):e71070. doi: 10.1002/hsr2.71070 (PMC12272301; doi:10.1002/hsr2.71070)
Supplement: Supplementary file 1 — S1 Vitamine D. [file HSR2-8-e71070-s001.pdf]

Estimation of Bioavailable Vitamin D

To assess BVD status, 5 ml of venous blood was collected from each participant under aseptic conditions and the direct supervision of the principal investigator. Samples were centrifuged within 30 minutes of collection to separate serum, which was then stored at −20°C until analysis. Due to the limited availability of BVP assays in clinical laboratories in Bangladesh, researchers used estimation formulas based on total 25(OH)D, albumin, and DBP. A serum total of 25(OH)D concentrations were measured using a chemiluminescence immunoassay (CLIA), and DBP levels were measured via ELISA. The equation we used is based on the established formula described by Bikle et al. (1986) and is expressed as:

$$\text{Free 25(OH)D} = \frac{\text{Total 25(OH)D}}{1 + (K_{\text{DBP}} \times [\text{DBP}]) + (K_{\text{Alb}} \times [\text{Albumin}])}$$

Where:

- $K_{\text{DBP}} = 6 \times 10^{8}\,\text{M}^{-1}$ is the binding affinity of 25(OH)D for DBP
- $K_{\text{Alb}} = 6 \times 10^{5}\,\text{M}^{-1}$ is the binding affinity of 25(OH)D for albumin
- Total 25(OH)D, DBP, and albumin concentrations were converted to molar units for calculation purposes

The blood test was tested at the Institute of Nuclear Medicine and Allied Science at DMCH and the Biochemistry Department. The level of BVD was categorized into the following groups: (Severe deficiency: less than 12 ng/ml; Moderate deficiency: 12 - 20 ng/ml; Insufficiency: between 21 - 29 ng/ml; Sufficient: higher than 30 ng/ml) (Acen et al., 2023). These categories are based on the concentration of 25(OH)D in the blood, which is the primary circulating form of vitamin D.

Assay characteristics for all three analytes: The following performance parameters were provided by the manufacturer and confirmed in-house:

- Total 25(OH)D: Sensitivity = 1.5 ng/ml; Intra-assay CV = 4.8%; Inter-assay CV = 6.1%
- DBP ELISA: Sensitivity = 1.0 µg/ml; Intra-assay CV = 5.2%; Inter-assay CV = 7.0%
- Albumin ELISA: Sensitivity = 0.1 g/dl; Intra-assay CV = 3.5%; Inter-assay CV = 5.6%

Supplementary Table: Component Variables Used to Calculate Bioavailable Vitamin D (BVD)

Without endometriosis

Participant ID	Total 25(OH)D (ng/ml)	DBP (µg/ml)	Albumin (g/dl)	Calculated BVD (ng/ml)
	19.6	281.8	3.8	19.3
2.	17.3	331.6	4.2	18.1
3.	17.4	286.1	3.9	18.9
4.	19.4	295.3	4.0	18.01
5.	20.4	293.9	4.1	22.3
6.	20.2	295.4	4.1	25.7
7.	23.1	280.5	4.3	29.5
8.	28.5	303.8	3.7	28.2
9.	24.4	303.6	3.6	29.1
10.	21.5	339.3	4.1	23.0
11.	12.1	331.1	4.0	15.1
12.	30.6	306.5	4.1	31.9
13.	22.4	323.3	4.4	25.01
14.	24.7	329.3	3.5	22.3
15.	23.4	300.3	3.6	23.6
16.	23.5	287.1	3.8	26.8
17.	29.5	305.5	3.9	31.8
18.	23.1	298.7	4.4	28.3
19.	29.7	322.6	3.7	23.01
20.	30.9	291.4	3.9	31.9
21.	34.8	324.8	4.1	33.01
22.	17.3	339.0	4.3	19.9
23.	13.4	282.2	3.8	15.6
24.	21.0	294.8	4.4	22.03
25.	23.1	302.0	4.2	24.9
26.	26.9	344.0	4.0	31.7
27.	24.0	297.3	4.1	25.9

28.	23.9	320.9	4.4	27.03
29.	30.0	295.4	4.5	31.7
30.	17.6	286.4	3.5	17.01
31.	12.6	323.9	4.3	14.9
32.	12.3	284.4	3.9	13.8
33.	25.9	317.0	4.5	27.08
34.	27.9	280.8	4.2	28.08
35.	28.2	317.6	3.8	29.07
36.	27.6	287.8	3.9	27.9
37.	24.8	346.8	4.4	25.1
38.	21.0	315.0	3.7	22.4
39.	31.7	340.9	3.8	33.6
40.	7.6	322.6	3.7	7.89
41.	11.4	317.8	4.3	13.8
42.	12.0	280.0	3.8	14.4
43.	13.3	345.0	4.4	15.01
44.	30.5	301.5	3.6	31.9
45.	26.2	346.3	3.6	27.03
46.	23.3	284.8	4.3	24.7
47.	38.5	289.0	4.0	38.42
48.	26.2	298.6	4.4	27.1
49.	24.3	294.8	4.0	25.3
50.	28.9	294.1	3.8	29.3
51.	21.9	325.5	3.9	22.05

With endometriosis

Participant ID	Total 25(OH)D (ng/mL)	DBP (µg/mL)	Albumin (g/dL)	Calculated BVD (ng/mL)
1.	13.8	288.5	3.7	14.1
2.	18.1	321.2	3.7	19.3
3.	15.3	285.0	4.1	17.05
4.	16.4	343.4	4.4	16.3
5.	7.4	296.7	4.2	7.4
6.	17.2	289.3	4.4	18.03
7.	13.6	313.1	4.3	15.04
8.	10.1	293.3	3.6	11.2
9.	13.5	309.7	4.0	14.03
10.	12.9	327.1	4.5	13.7
11.	10.5	308.2	3.8	11.5
12.	16.9	297.4	3.7	17.01
13.	16.7	309.5	3.8	10.9
14.	17.7	344.6	3.9	18.6
15.	13.9	318.5	3.6	15.7

16.	17.0	338.5	4.5	19.1
17.	21.9	339.4	3.7	22.7
18.	12.3	295.0	3.9	13.5
19.	10.9	306.5	4.5	10.1
20.	14.0	334.9	4.0	16.4
21.	10.3	347.0	4.5	10.7
22.	16.3	330.3	3.7	18.5
23.	13.5	347.8	4.1	14.5
24.	10.1	332.4	3.6	11.1
25.	14.8	315.2	4.4	15.6
26.	9.4	347.3	3.6	9.9
27.	16.8	321.7	4.2	17.5
28.	13.5	288.4	4.4	15.3
29.	10.7	321.6	4.1	11.7
30.	14.3	320.9	4.0	14.5
31.	18.0	294.3	4.2	19.1
32.	15.6	307.7	4.2	16.5
33.	11.5	302.1	4.3	11.1
34.	12.1	312.1	4.5	13.5
35.	17.9	285.1	3.7	18.05
36.	9.0	345.3	4.4	9.05
37.	10.2	305.9	3.7	10.1
38.	12.5	329.2	4.1	14.07
39.	14.8	325.8	3.5	16.03
40.	12.3	301.0	4.2	11.06
41.	14.1	289.4	3.6	17.5
42.	11.6	318.7	3.8	13.09
43.	17.1	330.2	3.7	11.05
44.	17.5	298.5	4.0	18.3
45.	12.6	339.2	3.6	13.9
46.	11.4	299.4	3.5	11.9
47.	13.6	324.6	3.8	15.06
48.	11.1	318.6	3.9	16.05
49.	10.1	285.3	4.4	10.1
50.	15.6	318.2	4.3	17.03

References

Acen, E. L., Worodria, W., Kateete, D. P., Olum, R., Joloba, M. L., Akintola, A., Bbuye, M., & Andia, I. B. (2023). Association of circulating serum free bioavailable and total vitamin D with cathelicidin levels among active TB patients and household contacts. *Scientific Reports*, *13*(1), 5365. https://doi.org/10.1038/s41598-023-32543-2

Bikle, D. D., Gee, E., Halloran, B., Kowalski, M. A., Ryzen, E., & Haddad, J. G. (1986). Assessment of the free fraction of 25-hydroxyvitamin D in serum and its regulation

by albumin and the vitamin D-binding protein. *J Clin Endocrinol Metab*, *63*(4), 954-959. https://doi.org/10.1210/jcem-63-4-954
